# Compound Dislocation of the First Phalanx of the Thumb

**Published:** 1842-05-21

**Authors:** E. Hartshorne

**Affiliations:** Resident Surgeon


### CLINICAL REPORTS.

### *Pennsylvania Hospital—Surgical Wards—Service of Dr. Norris.*

By E. HARTSHORNE, M. D., Resident Surgeon.

*Compound Dislocation of the first phalanx of the Thumb.*—N. L., drayman, ætat. 28, of temperate habits, while engaged in putting up his horse, allowed his thumb to be caught by the drawing chain, just as the horse was in the act of starting from the shafts of his dray.

The first phalanx of the left thumb was violently forced backwards, and the soft parts anterior to and more than half way around the joint, were torn open in such a manner as completely to expose the condyles of the second phalanx; which immediately presented through the wound, and in front of the articulating end of the first mentioned bone. With the parts in this condition he applied for admission into the hospital, an hour and a half after the occurrence of his mishap. Repeated and continued efforts at reduction, by means of the clove hitch and with the fingers, only subjected the man to great pain without avail. It was impossible to effect much extension on account of the extreme difficulty in retaining hold of the part to be extended.

The next morning, about sixteen hours after his entrance, the condyles of the second phalanx, comprising from three to four lines of its length, were excised with a small saw by Dr. Norris. The displaced bone was then restored to its natural position, the wound lightly dressed with adhesive plaster and patent lint, and the thumb extended at rest on a splint applied to the whole hand. The patient was restricted to a vegetable diet, and directed to keep his bed for a few days. The dressings were renewed on the third day, and every day thereafter throughout the treatment, the adhesive strip being early laid aside, and simple cerate dressing alone employed. No unhealthy inflammation or other unfavourable symptoms supervened ; and union, partially by granulation, steadily advanced with very little suppuration.

On the twenty-second day the splint was discontinued. Cicatrisation having been completed, further dressing was omitted on the thirty-first day. Slight motion could be produced at that time in the joint without causing much pain. The patient was discharged March 26, cured, with a slightly shortened but otherwise well shaped thumb.

*Remarks.*—The above is the second instance of this rare accident which has been presented in these wards within the last two years. The first was the case of a negro child about five years of age, in whom the injury resulted from a fall. The luxation was readily overcome by the attending surgeon, Dr. Randolph. The part was dressed as usual, and kept at rest in small finger splints. The little patient recovered in a short time with a good joint. In this instance the tender age of the individual considerably lessened the difficulty of the treatment.

In the other the subject was an adult, and the mechanical obstacles to the reduction appeared to be insuperable without an operation. These obstacles consisted in the locking of the prominent condyles of the respective bones, and the impossibility of keeping up forcible extension of the short and rounded disarticulated end without its slipping from the operator's grasp, whether he employed his own thumb and finger, or resorted to a form of the clove hitch. No attempt was made to enlarge the wound, which was already ample, or to divide the lateral ligaments. The removal of the small portion of the head of the second phalanx enabled the operator to effect the reduction immediately, and without pain ; while the edges of the wound were readily brought together, and so retained without subjecting the parts to any undue tension.

In page 96, No. 6 of the present volume of the Medical Examiner, will be found recorded a case similar to that now under consideration, occurring in a young man eighteen years of age, and not quite cured at the end of thirty-three days. The operators, however, were more fortunate in the youth of their patient, and in being able to preserve his joint by reduction easily performed without the aid of knife or saw. An abscess formed near the wound, but soon got well, having done no harm. The original articulation in N. L.'s case was of course destroyed ; but the degree of motion perceptible when the part was last examined, even then afforded strong reason to hope for the ultimate formation of a useful supplemental joint.

